# Factors associated with mortality in early stages of parkinsonism

**DOI:** 10.1038/s41531-022-00329-4

**Published:** 2022-06-02

**Authors:** Anouke van Rumund, Rianne A. J. Esselink, Marjolein B. Berrevoets-Aerts, Markus Otto, Bastiaan R. Bloem, Marcel M. Verbeek

**Affiliations:** 1 Department of Neurology, Donders Institute for Brain, Cognition and Behaviour, Radboud University Medical Center, Nijmegen, The Netherlands; 2 Radboudumc Center of Expertise for Parkinson & Movement Disorders, Nijmegen, The Netherlands; 3 Department of Neurology, Sint Anna Hospital, Geldrop, The Netherlands; 4 Department of Neurology, Ulm University Hospital, Ulm, Germany; 5 Department of Laboratory Medicine, Radboud University Medical Center, Nijmegen, The Netherlands

**Keywords:** Prognostic markers, Parkinson's disease

## Abstract

Prognosis of patients with parkinsonism varies greatly between the various parkinsonian syndromes. However, it is often difficult to distinguish the different forms, particularly in early stages. We examined predictors of mortality and functional outcome in patients with recent-onset parkinsonism with an initially uncertain diagnosis (*n* = 156). Patients were recruited between 2003 and 2006, comprehensively investigated, and followed prospectively (up to 15 years, mean 7 years). A final clinical diagnosis was established after follow-up. Independent predictors of mortality were investigated with multivariable Cox regression and combined into a simple prediction model. Model performance to predict 5- and 10-year mortality and functional outcome after 3 years was evaluated and externally validated in a second cohort of 62 patients with parkinsonism with an initially uncertain diagnosis. Ninety-one patients died (58%). Orthostatic hypotension, impaired cognition, abnormal tandem gait, and elevated neurofilament light chain concentration in serum or CSF were associated with mortality. A simple model that combined these factors showed excellent performance for prediction of functional outcome after 3 years and mortality after 5 and 10 years (*c*-statistic ~0.90 for all models). Model performance was confirmed after external validation: prediction of functional outcome after 3 years (*c*-statistic 0.89, 95% CI 0.80–0.98) and mortality after 5 years (*c*-statistic 0.91, 95% CI 0.84–0.99) were comparable to the results in the discovery cohort. These findings help clinicians to estimate a patient’s prognosis, irrespective of the specific diagnosis.

## Introduction

When patients present with new-onset parkinsonism in clinical practice, the initial focus is typically on diagnosing the disease, and then on treatment initiation. Additionally, offering reliable prognostic information is a crucial part of the initial patient counseling. Life expectancy is markedly reduced in persons with atypical parkinsonism (AP), such as multiple system atrophy (MSA), progressive supranuclear palsy (PSP), or corticobasal degeneration^1–4^. In contrast, Parkinson’s disease (PD) patients with normal cognition may have a largely normal life expectancy^3,5^. Moreover, AP patients are more likely to become wheelchair-bound or be admitted to a nursing home shortly after disease onset^1,2^. In these patients, timely identifying palliative care needs and initiating advanced care planning may be extra important^6^. However, particularly in early disease stages, it is often difficult to precisely diagnose the various parkinsonian syndromes, because of considerable overlap with the phenotype of PD. Misdiagnosis is common, with a diagnostic accuracy of only 58% in early stages of parkinsonism up till 85% when the diagnosis is made by a movement disorder expert^7,8^. When the diagnosis remains uncertain for a while, which is not rare, there is a risk of postponing the conversation about the patient’s prognosis because the patient remains in the “diagnostic process.“ Nonetheless, even when the clinical picture is ambiguous, a patient wishes to be informed as accurately as possible about the prognosis. Therefore, we evaluated which clinical signs, clinimetric scales, and biomarkers in serum and CSF could act as early predictors for survival and level of independence in a cohort of patients with an initially uncertain clinical diagnosis of parkinsonism at baseline. In this prospective observational cohort study, patients underwent extensive neurologic examination, laboratory testing and imaging, and also received prolonged follow-up, with extensive re-examination after 3 and 12 years. We aimed to develop and externally validate a model to predict mortality and functional status in patients with parkinsonism.

## Results

### Baseline characteristics and diagnosis

**Fig. 1:**
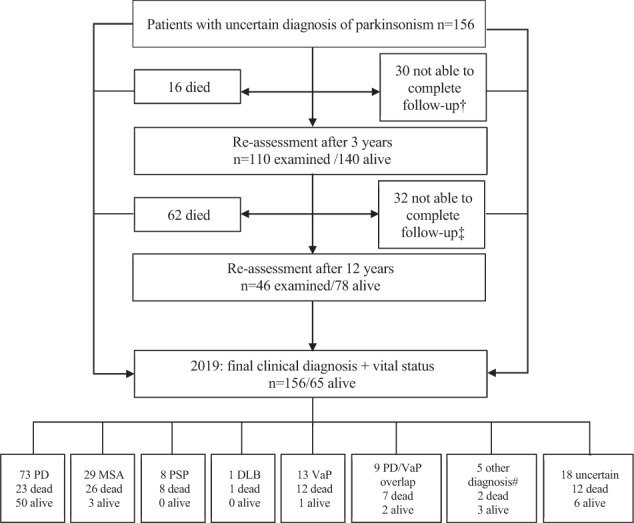
Flowchart of the study participants in the discovery cohort, follow-up, survival, and diagnoses. ^†^Follow-up information by telephone, survey, or medical chart (*n* = 25), lost-to-follow-up (*n* = 5). ^‡^Follow-up information by telephone, survey, or medical chart (*n* = 14), unable due to severe disease symptoms (*n* = 4), not willing to participate (*n* = 6), lost-to-follow-up (*n* = 9). ^#^Other diagnosis than neurodegenerative parkinsonism: idiopathic late onset cerebellar ataxia (*n* = 1), functional tremor (*n* = 1), medication-induced parkinsonism (*n* = 1), (stable) unilateral resting tremor without evidence of dopamine deficit on dopamine transporter imaging (*n* = 1), and superficial hemosiderosis due to trombocytopenia (*n* = 1). PD, Parkinson’s disease, MSA multiple system atrophy, PSP progressive supranuclear palsy, DLB dementia with Lewy bodies, VaP vascular parkinsonism.

Characteristics and candidate predictors of mortality in the discovery and validation cohort are shown in Tables 1 and 2. Median concentration of CSF neurofilament light chain (NFL) was higher in the validation cohort (2497 ng/L; elevated in 40%) compared to the discovery cohort (1580 mg/L, elevated in 28%). Other baseline characteristics were similar. Figure 1 demonstrates the flowchart of the study participants, including follow-up, survival, and diagnoses in the discovery cohort. Median follow-up duration was 7 years (interquartile range 2–12 years). One hundred and ten patients completed the clinical re-assessment after 3 years (out of 140 patients alive) and 46 patients completed the re-assessment after 12 years (out of 78 patients alive). At the end of the study when all 156 diagnoses were re-evaluated, 73 patients had a final diagnosis of PD, 29 MSA, 8 PSP, and 13 vascular parkinsonism (VaP). Nine patients had a clinical presentation suggestive of PD, but with unambiguous vascular abnormalities on MRI and were therefore classified as PD overlapping with vascular comorbidity. Most diagnoses were clinically based (97%), but there was neuropathologic confirmation in four cases (three MSA, one PSP). In one PD patient a Park2 mutation was found. These five pathologically or genetically confirmed diagnoses were all consistent with the final clinical diagnosis. In 18 patients (12%) the diagnosis of parkinsonism was still unclassifiable. In five patients an alternative diagnosis was made: idiopathic late onset cerebellar ataxia (*n* = 1), functional tremor (*n* = 1), medication-induced parkinsonism (*n* = 1), (stable) unilateral resting tremor without evidence of dopamine deficit on dopamine transporter imaging (*n* = 1), and superficial hemosiderosis due to trombocytopenia (*n* = 1).

**Table 1:** Characteristics of discovery and validation cohort.

	Missing,^†^ *n* (%)	Discovery cohort	
Patients, *n* (%)	0 (0%)	156	62
Mean age, years	0 (0%)	62 ± 10^#,PD,*^	64 ± 8
Gender, men (%)	0 (0%)	100 (64%)	40 (65%)
Mean age at symptom onset, years	0 (0%)	59 ± 10^#,PD^	61 ± 8
Symptom duration, years	0 (0%)	2 (2–4)	2 (1–3)
Poor functional outcome < 3, years	5 (3%)	60 (38%)	25 (40%)
Dead < 5 years	0 (0%)	32 (21%)	17 (27%)
Dead < 10 years	0 (0%)	70 (45%)	–
Mean survival (censored 12–2019), years	0 (0%)	10 ± 5	6 ± 3
Dead at end of follow-up, *n* (%)	0 (0%)	91 (58%)	21 (34%)
Mean age at death, years	0 (0%)	73 ± 8 (*n* = 91)	71 ± 7 (*n* = 21)

Values are means ± SD, medians (IQR), or *n* (%).

^†^In discovery cohort.

^#^Associated with survival (*p* < 0.05) by univariable Cox proportional hazard analysis.

^PD^Associated with survival in the PD subgroup (*p* < 0.05) by univariable Cox proportional hazard analysis.

^*^Selected for multivariable Cox proportional hazard analysis.

**Table 2:** Possible predictors of survival in discovery and validation cohort.

	Missing,^†^ *n* (%)	Discovery cohort	Validation cohort
Patients, *n* (%)	0 (0%)	156	62
Clinical characteristics			
Hyposmia	15 (10%)	42 (30%)^PD^	
Dysphagia	0 (0%)	46 (30%)^#^	
Urge incontinence	0 (0%)	38 (24%)^#^	
Orthostatic hypotension	8 (5%)	24 (16%)^#,PD^^,*^	8 (13%)
Depression	1 (1%)	60 (39%)	
Cognitive impairment (MMSE < 26)	0 (0%)	24 (15%)^#.PD.*^	13 (21%)
Tremor	0 (0%)	92 (59%)	
Falling	0 (0%)	41 (26%)^#^	
Abnormal tandem gait	2 (1%)	51 (33%)^#,PD,*^	26 (42%)
Ability to cycle	11 (7%)	91 (63%)^#,PD,*^	
Use of walking aid	2 (1%)	29 (19%)^#^	
Clinimetric scales			
Hoehn and Yahr score	0 (0%)	3 (2–3)^#^	2 (2–3)
UPDRS III score	4 (3%)	29 (19–37)^#,PD^	33 (26–43)
MMSE	5 (3%)	29 (27–30)^#,PD^	28 (27–30)
CSF and serum biomarkers			
CSF leukocytes, count/μL	7 (4%)	1 (1–2)	
CSF α-synuclein, μg/L	7 (4%)	25 (18–33)	
CSF Aβ_42_, ng/L	7 (4%)	811 (702–965)	
CSF phosphorylated tau, ng/L	7 (4%)	49 (37–59)	
CSF total tau, ng/L	7 (4%)	210 (159–293)^#^	
CSF NFL, ng/L	13 (8%)	1580 (968–3040) ^#,PD,*^	2497 (1283–4293)
Serum NFL, ng/L	24 (15%)	15 (9–25)^#,PD,*^	

Values are means ± SD, medians (IQR) or numbers (%).

MMSE Mini Mental State Examination, NFL neurofilament light chain, PD Parkinson’s disease, UPDRS III Unified Parkinson’s Disease Rating Scale, part III is the motor function subscale.

^†^In discovery cohort.

^#^Associated with survival (*p* < 0.05) by univariable Cox proportional hazard analysis.

^PD^Associated with survival in PD subgroup (*p* < 0.05) by univariable Cox proportional hazard analysis.

*Selectable Cox proportional hazard analysis.

### Mortality

Ninety-one (58%) of the 156 included patients with initially unclassifiable parkinsonism died during the study course (32% of PD patients, 90% of MSA, 100% of PSP, and 92% of VaP patients, shown in Fig. 1). Mean age at death was 73 years (76 years for PD patients, 70 years for patients with any form of AP). In 56% cause of death was related to parkinsonism (e.g., pneumonia, dementia, delirium, falls in advanced disease stage). In 12% there was a known other cause of death, not related to the parkinsonism (e.g., cancer (7%), cardiovascular disease such as heart infarction or stroke (3%), or other causes (2%)). In 32% cause of death was unknown. Because the follow-up length was substantially shorter in the validation cohort, fewer patients had deceased during the study course than in the discovery cohort (34%).

### Functional outcome

Three years after inclusion 60 (38%) patients had a poor functional outcome (14 patients were wheelchair-bound, 14 dependent on professional home care, 16 admitted to a nursing home, 45 were very dependent in their activities of daily living (Schwab & England Scale <50%). Ninety-one patients (58%) had a good functional outcome and five patients (3%) were lost to follow-up.

### Predictors of mortality

In multivariable analysis age, orthostatic blood pressure drop, cognitive impairment, abnormal tandem gait, and the biochemical marker NFL (both in CSF and serum) independently predicted mortality (Table 3). The results with multiple imputation of missings versus complete-case analysis were comparable. CSF and serum NFL were strongly correlated (Spearman’s correlation coefficient 0.80, *p* < 0.001). The predictive value of CSF NFL was slightly better, but since serum is more easily obtained than CSF, the multivariable model was evaluated with both CSF and serum NFL separately.

**Table 3:** Predictors of mortality in uncertain cases of (new-onset) parkinsonism.

	Survival model with CSF NFL	Survival model with serum NFL	
Predictors	HR (95% CI)	HR (95% CI)	
Age	1.05 (1.03–1.08)	1.05 (1.02–1.08)	
Orthostatic hypotension	3.28 (1.95–5.52)	2.51 (1.53–4.11)	
Abnormal tandem gait	1.91 (1.17–3.12)	2.68 (1.72–4.19)	
Cognitive impairment	1.71 (1.03–2.85)	1.82 (1.09–3.05)	
Elevated CSF NFL	3.29 (1.93–5.62)	–	
Elevated serum NFL	–	1.92 (1.15–3.18)	
	Prediction model with CSF NFL in discovery cohort	Prediction model with serum NFL in discovery cohort	Prediction model with CSF NFL in validation cohort
	*c*-statistic (95% CI)	*c*-statistic (95% CI)	*c*-statistic (95% CI)
10-year mortality	0.90 (0.88–0.92)	0.87 (0.85–0.89)	–
5-year mortality	0.90 (0.88–0.92)	0.88 (0.86–0.90)	0.91 (0.84–0.99)
3-year functional outcome	0.94 (0.92–0.95)	0.92 (0.90–0.94)	0.89 (0.80–0.98)

Predictive values in the model: age in years, orthostatic hypotension, abnormal tandem gait, cognitive impairment, elevated CSF NFL (>2700 ng/L), elevated serum NFL (>14.8 ng/L). *C*-statistics are calculated after correction for optimism. HR hazard ratio, CI confidence interval, MMSE Mini Mental State Examination, NFL neurofilament light chain.

### Prediction model

**Fig. 2:**
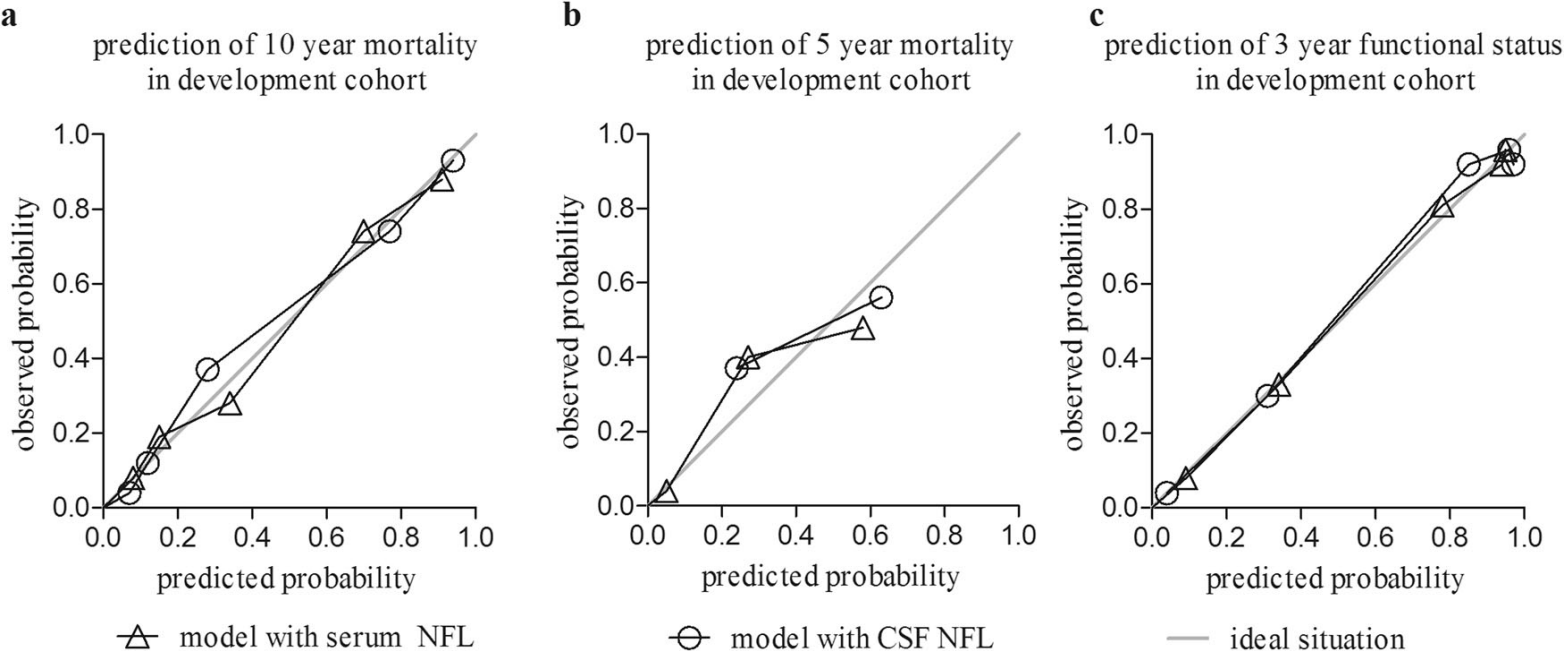
Calibration plots of the prediction model per outcome measure in the discovery cohort. Calibration plots of prediction models in the discovery cohort for prediction of (**a**) 10-year mortality, (**b**) 5-year mortality, and (**c**) 3-year functional outcome. The gray reference line represents the ideal line. Observed probabilities are indicated by quintiles of predicted probability.

A simple model based on the presence of the independent predictors of mortality (i.e., orthostatic hypotension, impaired cognition, abnormal tandem gait, and the biomarker NFL) had excellent *c*-statistics for the prediction of 5- and 10-year mortality and functional outcome after 3 years (Table 3). Calibration of the models was accurate (Fig. 2). In the subgroup of PD patients (*n* = 73), *c*-statistics of the models remained very good (between 0.87 and 0.96, Table 4). Regression equations of the various models are provided in the Supplementary Table. Table 5 shows a risk chart of estimated probabilities of an unfavorable outcome per outcome measure (i.e., 3-year functional outcome, 5 and 10-year mortality) based on the prediction model. Of the 80 patients without any clinical predictor present, 69% was still alive at the end of the follow-up period (in December 2019, 13–16 years after study entry) and 58% still lived independently at time of the 12-year clinical assessment. In contrast, half of the patients with two or more clinical predictors present had deceased in the first 5 years of follow-up. Especially when none or only one of the clinical signs was present, the biomarker NFL was of additional value to differentiate patients with a low versus intermediate and intermediate versus high risk of an unfavorable outcome.

**Table 4:** Predictors of mortality in Parkinson’s disease subgroup.

	Survival model with CSF NFL	Survival model with serum NFL
Predictors	HR (95% CI)	HR (95% CI)
Age	1.12 (1.04–1.20)	1.14 (1.05–1.23)
Orthostatic hypotension	3.24 (0.90–11.65)	3.04 (0.91–10.19)
Abnormal tandem gait	3.65 (0.87–15.36)	4.10 (1.24–13.57)
Cognitive impairment	5.84 (1.80–18.90)	5.82 (1.78–19.06)
Elevated CSF NFL^a^	2.29 (0.12–43.54)	–
Elevated serum NFL^b^	–	0.70 (0.22–2.25)
	Prediction model with CSF NFL in discovery cohort	Prediction model with serum NFL in discovery cohort
	*C*-statistic (95% CI)	*C*-statistic (95% CI)
10-year mortality	0.88 (0.84–0.92)	0.87 (0.83–0.91)
5-year mortality	0.96 (0.94–0.98)	0.96 (0.94–0.98)
3-year functional outcome	0.88 (0.83–0.94)	0.88 (0.82–0.93)

Predictive values in the model: age in years, orthostatic hypotension, abnormal tandem gait, cognitive impairment, elevated CSF NFL (>2700 ng/L), elevated serum NFL (>14.8 ng/L). HR hazard ratio, CI confidence interval, MMSE Mini Mental State Examination, NFL neurofilament light chain.

^a^One patient.

^b^Nine patients.

**Table 5:** Prediction chart of unfavorable outcome in patients with uncertain parkinsonism.

	Number of clinical predictors present			
	0	1	2	3
Wthout CSF NFL				
3-year functional outcome	18%	*59%*	**91%**	**99%**
5-year mortality	5%	14%	*33%*	*58%*
10-year mortality	27%	*57%*	**83%**	**96%**
Normal CSF NFL				
3-year functional outcome	5%	*37%*	**84%**	**98%**
5-year mortality	1%	5%	16%	*38%*
10-year mortality	12%	*38%*	**73%**	**93%**
Elevated CSF NFL				
3-year functional outcome	30%	**80%**	**97%**	**>99%**
5-year mortality	8%	23%	*50%*	**78%**
10-year mortality	*42%*	**76%**	**93%**	**99%**

The values <33%: low risk of unfavorable outcome. The values 33–66% (in italics): intermediate risk of unfavorable outcome. The values >67% (in bold): high risk of unfavorable outcome. Absolute probabilities of unfavorable outcome (i.e., poor functional outcome or dead in 5 or 10 years) in patients with recent-onset parkinsonism of uncertain etiology based on multivariable prediction model (see Table 3). This chart demonstrates the value of CSF NFL analysis additional to clinical evaluations. For example, a patient with abnormal score on only one of the clinical predictors (i.e., orthostatic hypotension, abnormal tandem gait or cognitive impairment) has an intermediate risk to become care-dependent within 3 years; however, when CSF NFL is elevated (>2700 ng/L) there is a high risk to become care-dependent within 3 years, in contrast to the intermediate risk when CSF NFL levels are normal.

### External validation of the model

External validation of the models showed a *c*-statistic of 0.91 (95% CI 0.84–0.99) for prediction of 5-year mortality and 0.89 (95% CI 0.80–0.98) for prediction of functional outcome after 3 years (Table 3).

## Discussion

In this prospective longitudinal cohort study, we evaluated survival and mortality of patients with recent-onset parkinsonism but with an uncertain diagnosis, based on clinical grounds, at the time of inclusion. We found that mortality was associated with three simple clinical signs plus a biomarker: (1) presence of orthostatic hypotension, (2) impaired cognition, (3) impaired tandem gait, and (4) increased NFL in either serum or CSF. In addition, these predictors could be used to predict functional outcome after 3 years and mortality after 5 and 10 years with excellent model performance and with external validation of the models. Moreover, the prediction models performed similarly when only PD patients were considered. We will next discuss the predictors and prediction models in further detail.

Orthostatic hypotension is independently associated with mortality in the elderly and is associated with falls and cardiovascular events^9,10^. In patients with parkinsonism, the presence of orthostatic hypotension in an early disease phase likely reflects underlying disorders with a more aggressive course, including MSA and dementia with Lewy bodies. Our present findings are in keeping with early orthostatic hypotension having a poor prognostic value.

Older age at the onset and presence of dementia are the most consistently reported independent predictors of mortality in PD^5^, which was confirmed in our study. In line with this, a recent Swedish population cohort study of patients with parkinsonism showed that PD patients with normal cognitive function had a fairly normal life expectancy^3^. This corresponds with our finding that the absence of the clinical predictors (including the absence of cognitive impairment) is associated with a long survival time and preserved independence.

An abnormal tandem gait is likely an early indicator of postural instability and resultant falls^11^, both being major factors determining quality of life, morbidity, and mortality in parkinsonism^12,13^. Furthermore, an abnormal tandem gait is a red flag signaling the presence of an underlying AP, which in turn is associated with a markedly increased mortality risk^11^.

NFL is a protein constituent of myelinated axons and is released in the extracellular space as a consequence of neurodegeneration or axonal injury^14^. Previous publications, including reports from our group, showed that an elevated level of the biomarker NFL, both in serum and CSF, can discriminate PD from AP^15–18^. A prospective follow-up study found that plasma NFL levels correlated with disease severity and progression in terms of both motor and cognitive functions in PD^19^. In line with this, NFL, both in serum and CSF, was associated with high mortality and poor functional outcome in our study. High levels of NFL likely reflect a more widespread and aggressive underlying process of neurodegeneration.

We demonstrated that patients with two or more of the above-mentioned clinical signs are likely to have an unfavorable disease course; this included a high risk of becoming wheelchair-bound, care-dependent and/or institutionalized in the next 3 years and a small chance of survival after 10 years (Table 5). For these patients advanced care planning might be warranted. When none or only one of the clinical signs is present, the biomarker NFL can be of additional value to differentiate patients with a low versus intermediate and intermediate versus high risk of an unfavorable outcome. Patients with normal cognition, tandem gait, and orthostatic blood pressure in combination with a normal NFL concentration in CSF or serum are likely to have a milder disease course, whereas an increased NFL is associated with an increased risk for an unfavorable outcome in these patients.

The risk chart presented in this study can contribute to better, patient-tailored, prognostic information for patients with recent-onset parkinsonism. Since symptoms of PD and AP may overlap, particularly in early disease stages when misdiagnoses are common^7,8^, these findings may help clinicians to better estimate a patient’s prognosis, irrespective of the specific diagnosis.

The strongest point of this study is the inclusion of two unique cohorts of patients with an uncertain diagnosis. The patients in both cohorts have been studied in detail at baseline and were followed up, in case of the discovery cohort, for even more than 12 years. Previous studies typically included patients in whom the diagnosis was already clear. However, in daily clinical practice, many patients present with an initially uncertain diagnosis, particularly when patients are first seen by a physician who is not deeply experienced in movement disorders. Our study now provides guidance for counseling of such patients with respect to the prognosis, and specifically in relation to survival and level of dependence. The simple prediction model has the potential to help the clinician to assess the patient’s prognosis even at first visit and at an early disease stage when there is still a lot of uncertainty about the diagnosis.

This study also has limitations. First, mortality and functional outcome are not the only relevant parameters for the overall prognosis in patients with parkinsonism. For instance, predictors of quality of life were not evaluated in this study. Second, the underlying diagnosis of PD or a form of AP (which was initially undetermined in these patients) was certainly an important factor associated with survival time. It is well known that patients with AP have a markedly worse prognosis. Therefore, the predictors that we identified as being associated with survival were probably also indirect markers for the underlying diagnosis. The survival analyses were not adjusted for the ultimate diagnosis made after follow-up, since only baseline factors were included and the diagnosis was ambiguous at baseline. However, the predictive factors in the final multivariable prediction models remained significant (at the 0.001 level) after adjustment for the final clinical diagnosis and the *c*-statistics of the models was still very good in the subgroup of PD patients. Moreover, we aimed specifically for a prediction model regardless of the specific diagnosis at baseline, because this diagnosis is often unclear or uncertain in an early stage. In some cases the diagnosis remains uncertain, even during follow-up, as occurred in 12% of the patients in our discovery cohort (Fig. 1). Third, the results may not be generalizable to all patients with parkinsonism, because the patients in both the discovery and validation cohorts were recruited from a specialized movement disorders clinic (with possible underrepresentation of older patients and milder cases). Both cohorts comprised patients with an initial ambiguous clinical presentation and excluded patients with a full-blown clinical picture. Nonetheless, we believe that in this population in particular (i.e., uncertain cases with an ambiguous clinical presentation) it is most difficult to estimate prognosis. In these cases, extra tools to predict disease course and outcome would be helpful for proper counseling. To recognize clinical and laboratory features associated with mortality and care dependency could help the clinician in counseling their patients. Moreover, since the factors in our study (orthostatic hypotension, balance problems, cognitive decline) are also associated with mortality in the elderly population in general, we expect that they could well apply to a wider range of parkinsonism patients; however, our cohorts were not suitable to test this hypothesis. Finally, sample size in the validation cohort was relatively small; ~40% of patients in the second cohort was excluded from the prediction model because follow-up was less than 5 years yet or there was no CSF obtained. Nonetheless, model performance was very good and significant. Unfortunately, serum NFL was not available in the validation cohort.

Further external validation of the prediction models and risk chart in different prospective cohorts might gain further insight in the robustness of the model and generalizability. If robustness is confirmed, this model can contribute to estimating the prognosis and incorporating this into patient-tailored information for those with new-onset parkinsonism. The three clinical factors associated with mortality (orthostatic blood pressure, cognition, and tandem gait) are all simple to assess in the consulting room. The risk chart can help a clinician decide whether an ancillary test of the biomarker NFL (in serum or CSF) has additional prognostic value. Finally, although not evaluated here, early recognition and treatment of the clinical factors that predicted mortality in this study, particularly orthostatic hypotension and impaired balance, may even prevent or postpone morbidity and mortality related to these factors.

## Methods

### Research questions

Our primary research question was: what are predictors of mortality in patients with recent-onset parkinsonism for whom, because of an atypical or not fully matured presentation, no specific diagnosis (PD or a form of AP) could as yet be established? The secondary research question was whether these predictors of mortality could also predict functional outcome (i.e., level of independence) 3 years after inclusion.

### Standard protocol approvals and patient consent

This study was approved by the ethical committee review board Arnhem-Nijmegen in The Netherlands and was performed in accordance with the Declaration of Helsinki. All participants provided written informed consent for participation.

### Study participants

All participants were part of a previously described prospective study performed at the Radboud University Medical Center (Nijmegen, The Netherlands)^20^. Patients were consecutively recruited from our movement disorders outpatient clinic between January 2003 and December 2006. All participants had clear signs of parkinsonism, but with an uncertain specific diagnosis at the time of inclusion. Uncertainty was defined as uncertainty about the specific form of parkinsonism according to expert opinion of the movement disorder specialist after the first visit (e.g., because of an atypical or not fully matured presentation, or presence of red flags for a diagnosis of PD, but with insufficient ground to diagnose a specific form of AP). Exclusion criteria were age under 18 years, history of brain surgery or other neurodegenerative disease than parkinsonism, and unstable comorbidity. All patients underwent a structured interview, detailed and standardized neurological examination and, within 6 weeks after the initial visit, blood collection and lumbar puncture among other ancillary investigations (brain MRI, ^123^I-iodobenzamide-SPECT, anal sphincter EMG). The study design, methods, and included patient populations have been described extensively^20^.

### Sample size

The required sample size for the analysis was calculated to evaluate whether the size of the development cohort (*n* = 156) was sufficient for the (time-to-event) analysis. Based on a (Cox–Snell) *R*^2^ value of 0.50, a mortality rate of 45% in the cohort during follow-up, six candidate predictors for potential inclusion in the prediction model and a shrinkage factor of 0.9 to correct for optimism in the model, a minimum sample size of 148 was required^21,22^.

### Clinical assessment

Standardized clinical assessments were performed at baseline. In a structured interview 38 items were documented (e.g., absence/presence of hyposmia, falls, depression); during an extensive neurological examination 30 items were examined (e.g., tremor, balance, orthostatic hypotension)^23^. Orthostatic hypotension was defined as orthostatic decrease of blood pressure within 3 min of standing by at least 30 mmHg systolic or 15 mmHg diastolic, in the absence of dehydration, medication, or other diseases that could plausibly explain autonomic dysfunction. Tandem gait was scored as normal when a patient could walk 10 consecutive steps on a line without or with only one side step, and was scored abnormal if the patient had two or more side steps or was unable to perform the task^11^. Clinimetric scales included the Hoehn and Yahr (H&Y) score, Unified Parkinson’s Disease Rating part III (UPDRS III) motor score, International Cooperative Ataxia Rating Scale (ICARS), Schwab and England scale and Mini mental state examination (MMSE, a total score <26 indicated cognitive impairment)^24–27^. Surviving patients had routine clinical follow-up. The same standardized and extensive clinical assessments as during the baseline visit were repeated after 3 and 12 years.

### Serum and CSF biomarkers

Serum and CSF samples were collected in polypropylene tubes, centrifuged, aliquoted, and stored in polypropylene tubes at −80°C until analysis. Laboratory technicians blinded for clinical symptoms and outcome performed all serum and CSF analyses. The following CSF variables were analyzed: α-synuclein real-time quaking-induced conversion (α-syn RT-QuIC), amyloid β42 (Aβ42), total tau protein (t-tau), tau protein phosphorylated at Thr181 (p-tau), α-synuclein, neurofilament light chain (NFL), 3-methoxy-4-hydroxyphenylethyleneglycol (MHPG), 5-hydroxyindolacetic acid (5-HIAA), homovanillic acid (HVA), blood pigments, and the total cell count. CSF analyses were performed as previously described^20^. Serum NFL was measured with single molecule array (Simoa) as described elsewhere^15,28^. For CSF NFL, a concentration of >2700 ng/L was used as cutoff, and for serum NFL a concentration of >14.8 ng/L, based on optimal cutoff values for discrimination of PD, APD, and controls as previously published^15,16^.

### Diagnosis

After 3 years of follow-up, a clinical diagnosis was established in consensus by two movement disorder specialists according to the clinical criteria that existed at the time^20,29–32^ and was based on evolution of the clinical presentation (including rate of progression, and possibly emergence of red flags signaling presence of AP), MRI findings (at baseline), and response to dopaminergic therapy (both efficacy and tolerance). Twelve years after inclusion, all clinical diagnoses were evaluated again and updated according to the most recent clinical criteria^33–35^, the disease course from study entry until latest clinical follow-up, response to dopaminergic therapy, and, whenever available, neuropathologic confirmation.

### Survival

The primary outcome was all-cause mortality. Information on the vital status and date of death of participants was retrieved from the Dutch Municipal Personal Records database (December 2019). Cause of death was retrieved from the medical charts. Survival duration was calculated from study entry until date of death and censored at December 1, 2019. We deliberately measured mortality and survival from study entry (i.e., time since diagnosis of parkinsonism of uncertain etiology, shortly after the first visit in our movement disorder clinic) rather than from symptom onset or diagnosis, for the following reasons: (1) patients’ recall of symptom onset is often unreliable and (2) critically important, survival from symptom onset overestimates the time at risk as no deaths in patients will be detected before diagnosis (only alive patients are diagnosed and included in the study, introducing an immortal time bias), thus underestimating mortality.

### Functional status after 3 years

The secondary outcome was functional status (level of independence) at the standardized assessment 3 years after inclusion. A poor functional status was scored for patients who were either wheelchair-bound or had substantial difficulties to perform activities of daily living (i.e., “very dependent” according to the Schwab and England scale <50%), or were dependent on professional home care or admitted to a nursing home. A good functional status was scored for patients without these characteristics. If patients were unable to participate in the assessment after 3 years (because the symptoms had become too severe, or when they had deceased), functional outcome was also scored as “poor”.

### Statistical analyses

Data analyses were done using IBM SPSS Statistics 22 (Armonk, NY, USA) and GraphPad Prism 5 (La Jolla, CA, USA). Baseline characteristics were compared between alive and deceased patients in December 2019 using Student’s *t* test, Mann–Whitney *U* test or *χ*^2^ test whenever appropriate. Correlations between two variables were investigated by Pearson’s or Spearman’s test as appropriate. Cox proportional hazard analysis was used to investigate which (1) clinical signs, (2) clinimetric scales, and (3) serum and CSF biomarkers predicted mortality. Because age is strongly related to survival, all results were adjusted for age at inclusion. Those baseline clinical signs, clinimetric scales, and biomarkers significantly associated with survival at the *p* < 0.05 level in the univariate models were included in a multivariable model using a backward elimination procedure. Given the one-in-ten rule with one predictive variable for every ten outcome events, we could include a maximum of six predictors in the multivariable analysis. Missing values were evaluated with Little’s Missing Completely at Random (MCAR) test and fulfilled the significance level >0.05. Missing values in the discovery cohort were imputed with multiple imputation (5 iterations). Two variables, ICARS and CSF α-syn RT-QuIC, had many missing values (>15%) and were therefore excluded from the analysis. If variables were highly correlated (*r* > 0.70), the variable with the lowest *p* value was included to avoid colinearity. Adjusted hazard ratios (aHRs) and 95% confidence intervals (CI) were calculated. The proportional hazard assumption was evaluated using Schoenfeld residuals^36^.

A simple prediction model was developed based on the best predictors of mortality from the multivariable Cox proportional hazard analysis. Subsequently, this model was used to predict functional outcome after 3 years and mortality after 5 and 10 years. Predicted probabilities for the different outcome measures were calculated with binomial logistic regression. To minimize overfitting of predictor effects, we internally validated the initial models by performing bootstrapping (>2000 bootstrap samples). A shrinkage factor of 0.9 was estimated from the bootstrap procedure and regression coefficients in the models were multiplied by this shrinkage factor to correct for overfitting. Model performance was assessed with discrimination (with the *c*-statistic) and calibration (with calibration plots). A schematic representation of the model development steps is provided as a supplemental figure with the article.

### External validation

For external validation of the prediction model, we used a second cohort of 62 different patients with parkinsonism of uncertain etiology, recruited from our outpatient movement disorder clinic between 2010 and 2017. Patients underwent standardized clinical assessment, lumbar puncture, and MRI as described elsewhere^23^. The original cohort comprised 105 patients, but only patients with complete data regarding the predictors and sufficient follow-up length to evaluate 5-year mortality were included. In this cohort, follow-up length was not yet sufficient to evaluate 10-year mortality. The prediction model was applied with the biomarker NFL in CSF, since serum NFL was not assessed in this cohort. Model performance to predict 3-year functional status and 5-year mortality was assessed with the *c*-statistic. Results are reported in accordance with the Transparent Reporting of a multivariable prediction model for Individual Prognosis or Diagnosis statement^37^.

### Reporting summary

Further information on research design is available in the Nature Research Reporting Summary linked to this article.

## Supplementary information

Supplemental material

Reporting Summary

## Data Availability

The clinical data described in this manuscript are stored and can be found at the department of Neurology of the Radboud University Medical Centre in Nijmegen, The Netherlands. The biological specimens (CSF and serum samples) are stored at the department of Laboratory Medicine, Radboud University Medical Centre in Nijmegen, The Netherlands. The data are documented in Dutch or English, according to the FAIR principles. Requests for data sharing should be sent to the corresponding author and will be shared after approval by the co-authors.

## Code availability

Regression equations for the models in the discovery cohort are provided in a supplementary table with the article.
