# Supplementary material for: Factors associated with mortality in early stages of parkinsonism
Source: NPJ Parkinsons Dis. 2022 Jun 2;8:67. doi: 10.1038/s41531-022-00329-4 (PMC9163117; doi:10.1038/s41531-022-00329-4)
Supplement: Supplementary file 1 — Supplemental material [file 41531_2022_329_MOESM1_ESM.docx]

Supplementary figure. Schematic representation of model development steps.

**Selection of candidate predictors**

Univariable analysis adjusted for age (n=22)

Selection of a maximum of 6 predictors

**Missings**

Multiple imputation of missings (5 iterations)

**Multivariable model**

Multivariable Cox proportional hazard analysis

Stepwise backward elimination

**Prediction models**

10 year mortality, 5 year mortality

3 year functional outcome

**Correction for optimism / overfitting**

Bootstrapping (>2000) of all models

Shrinkage factor x regression coefficients

**Model performance**

Internal: C-statistic, calibration plots

External: secondary cohort

**Model presentation**

Risk chart

Supplementary table. Regression equations with intercepts and regression coefficients of the models

| **Regression equations for the models with CSF NFL** | |
| --- | --- |
| **10 year mortality** | -5.00061+0.047934*AGE+1.864858*ORTHOSTATIC HYPOTENSION+1.17738*ABNORMAL TANDEM GAIT+1.512675*COGNITIVE IMPAIRMENT+1.688653*ELEVATED CSF NFL |
| **5 year mortality** | -7,57827+0.054164*AGE+1.49344*ORTHOSTATIC HYPOTENSION+1.404595*ABNORMAL TANDEM GAIT+0.852008* COGNITIVE IMPAIRMENT+1.730294*ELEVATED CSF NFL |
| **3 year functional outcome** | -3.82211+0.014932*AGE+1.971458*ORTHOSTATIC HYPOTENSION+1.800583*ABNORMAL TANDEM GAIT+3.23842*COGNITIVE IMPAIRMENT+2.051318*ELEVATED CSF NFL |
