# Supplementary material for: Factors associated with mortality in early stages of parkinsonism
Source: NPJ Parkinsons Dis. 2022 Jun 2;8:67. doi: 10.1038/s41531-022-00329-4 (PMC9163117; doi:10.1038/s41531-022-00329-4)
Supplement: Supplementary file 2 — Reporting Summary [file 41531_2022_329_MOESM2_ESM.pdf]

nature portfolio

Corresponding author(s): A. van Rumund

Last updated by author(s): Apr 4, 2022

Reporting Summary

Nature Portfolio wishes to improve the reproducibility of the work that we publish. This form provides structure for consistency and transparency in reporting. For further information on Nature Portfolio policies, see our Editorial Policies and the Editorial Policy Checklist.

Statistics

For all statistical analyses, confirm that the following items are present in the figure legend, table legend, main text, or Methods section.

n/a	Confirmed	
☐	☒	The exact sample size (n) for each experimental group/condition, given as a discrete number and unit of measurement
☐	☒	A statement on whether measurements were taken from distinct samples or whether the same sample was measured repeatedly
☐	☒	The statistical test(s) used AND whether they are one- or two-sided<br>*Only common tests should be described solely by name; describe more complex techniques in the Methods section.*
☐	☒	A description of all covariates tested
☐	☒	A description of any assumptions or corrections, such as tests of normality and adjustment for multiple comparisons
☐	☒	A full description of the statistical parameters including central tendency (e.g. means) or other basic estimates (e.g. regression coefficient) AND variation (e.g. standard deviation) or associated estimates of uncertainty (e.g. confidence intervals)
☐	☒	For null hypothesis testing, the test statistic (e.g. F, t, r) with confidence intervals, effect sizes, degrees of freedom and P value noted<br>*Give P values as exact values whenever suitable.*
☒	☐	For Bayesian analysis, information on the choice of priors and Markov chain Monte Carlo settings
☐	☒	For hierarchical and complex designs, identification of the appropriate level for tests and full reporting of outcomes
☐	☒	Estimates of effect sizes (e.g. Cohen's d, Pearson's r), indicating how they were calculated

Our web collection on statistics for biologists contains articles on many of the points above.

Software and code

Policy information about availability of computer code

Data collection	no software was used
Data analysis	Data analyses were done using IBM SPSS Statistics 22 (Armonk, NY, USA) and GraphPad Prism 5 (La Jolla, CA, USA).

For manuscripts utilizing custom algorithms or software that are central to the research but not yet described in published literature, software must be made available to editors and reviewers. We strongly encourage code deposition in a community repository (e.g. GitHub). See the Nature Portfolio guidelines for submitting code & software for further information.

Data

Policy information about availability of data

All manuscripts must include a data availability statement. This statement should provide the following information, where applicable:

- Accession codes, unique identifiers, or web links for publicly available datasets
- A description of any restrictions on data availability
- For clinical datasets or third party data, please ensure that the statement adheres to our policy

The clinical data described in this manuscript are stored and can be found at the department of Neurology of the Radboud University Medical Centre in Nijmegen, The Netherlands. The biological specimens (CSF and serum samples) are stored at the department of Laboratory Medicine, Radboud University Medical Centre in Nijmegen, The Netherlands. The data are documented in Dutch or English, according to the FAIR principles. Requests for data sharing should be sent to the corresponding author and will be shared after approval by the co-authors. Regression equations for the models in the discovery cohort are provided in a supplementary table with the article.

Field-specific reporting

Please select the one below that is the best fit for your research. If you are not sure, read the appropriate sections before making your selection.

☒ Life sciences ☐ Behavioural & social sciences ☐ Ecological, evolutionary & environmental sciences

For a reference copy of the document with all sections, see nature.com/documents/nr-reporting-summary-flat.pdf

Life sciences study design

All studies must disclose on these points even when the disclosure is negative.

Sample size	We used a discovery cohort of 156 participants and a validation cohort of 62 participants. Regarding the cohort sizes, no formal sample size calculation was done prior to this study. However, regarding the multivariable models, the number of variables was based on the number of outcome events, which allowed a maximum of 6 variables in the analysis.
Data exclusions	Only clinical data and biomarker results were included in the analysis. Participants of the study cohort also underwent 123I-iodobenzamide-SPECT and anal sphincter EMG, but these results were excluded from the analysis since they seemed unrelated to this particular research question (and survival).
Replication	For external validation of the prediction model, we used a second cohort of 62 different patients with parkinsonism of uncertain etiology, recruited from our outpatient movement disorder clinic between 2010 and 2017. The original cohort comprised 105 patients, but only patients with complete data regarding the predictors and sufficient follow-up length to evaluate 5-year mortality were included.
Randomization	not applicable; participants were not allocated to experimental groups because no intervention was studied, therefore randomization did not take place.
Blinding	not applicable; clinical data and biomarker results were obtained before survival data were obtained and disclosed.

Reporting for specific materials, systems and methods

We require information from authors about some types of materials, experimental systems and methods used in many studies. Here, indicate whether each material, system or method listed is relevant to your study. If you are not sure if a list item applies to your research, read the appropriate section before selecting a response.

Materials & experimental systems

n/a	Involved in the study	
☒	☐	Antibodies
☒	☐	Eukaryotic cell lines
☒	☐	Palaeontology and archaeology
☒	☐	Animals and other organisms
☐	☒	Human research participants
☐	☒	Clinical data
☒	☐	Dual use research of concern

Methods

n/a	Involved in the study	
☒	☐	ChIP-seq
☒	☐	Flow cytometry
☒	☐	MRI-based neuroimaging

Human research participants

Policy information about studies involving human research participants

Population characteristics	The study population consisted of 156 patients with parkinsonism in the discovery cohort and 62 patients in the validation cohort. All participants had clear signs of parkinsonism, but with an uncertain specific diagnosis at the time of inclusion. Uncertainty was defined as: uncertainty about the specific form of parkinsonism according to expert opinion of the movement disorder specialist after the first visit (e.g. because of an atypical or not fully matured presentation, or presence of red flags for a diagnosis of PD, but with insufficient ground to diagnose a specific form of AP). Exclusion criteria were age under 18 years, history of brain surgery or other neurodegenerative disease than parkinsonism, and unstable comorbidity.
Recruitment	Patients were consecutively recruited from our movement disorders outpatient clinic between January 2003 and December 2006 (discovery cohort) or, for the validation cohort, between 2010 and 2017. Since both cohorts were recruited in a specialized movement disorder clinic (with possible underrepresentation of older patients and milder cases), the results may not be generalizable to all patients with parkinsonism, as discussed in the discussion section.
Ethics oversight	This study was approved by the ethical committee review board Arnhem-Nijmegen in The Netherlands and was performed in accordance with the Declaration of Helsinki. All participants provided written informed consent for participation.

Note that full information on the approval of the study protocol must also be provided in the manuscript.

Clinical data

Policy information about clinical studies

All manuscripts should comply with the ICMJE guidelines for publication of clinical research and a completed CONSORT checklist must be included with all submissions.

Clinical trial registration	this study is an observational study and no clinical trial. It was therefore not registered as clinical trial.
Study protocol	https://doi.org/10.1007/s00415-014-7568-4
Data collection	January 2003 - December 2006 (discovery) and January 2011 - December 2017 (validation) Radboudumc Center of Expertise for Parkinson & Movement Disorders, Nijmegen, The Netherlands.
Outcomes	Our primary research question was: what are predictors of mortality in patients with recent onset parkinsonism for whom, because of an atypical or not fully matured presentation, no specific diagnosis (PD or a form of AP) could as yet be established? The secondary research question was whether these predictors of mortality could also predict functional outcome (i.e. level of independence) three years after inclusion.
